# A Mouse Model of Cancer Induced Bone Pain: From Pain to Movement

**DOI:** 10.3389/fnbeh.2022.873750

**Published:** 2022-06-23

**Authors:** Haiwang Ji, Xiang Jin, Qing Zhang, Yuan Zhou, Chan Zhu, Yan Yang, Zongxiang Tang, Guang Yu, Changming Wang

**Affiliations:** School of Medicine and Holistic Integrative Medicine, Nanjing University of Chinese Medicine, Nanjing, China

**Keywords:** CIBP, motor coordination, locomotion, pain, patellar ligament

## Abstract

Cancer induced bone pain (CIBP) occurs in patients with advanced osteosarcoma or metastasized bone tumors that can negatively affects the patient's quality of life. However, motor impairment in CIBP is still understudied. To improve the quality of life of patients with CIBP, the study of CIBP induced movement impairment is of particular importance. Here, we presented a model of metastatic cancer induced bone pain caused by an allograft of Lewis lung cancer cells. In this method, we injected Lewis lung cancer cells into the femoral medulla cavity and recorded the pain behavior and motor behavior after CIBP surgery. We observed enhanced pain after the initial surgery. Interestingly, we found the latency on rotarod was significantly reduced concomitant with tumor growth and pain. This result indicated that the motor coordination and balance were severely impaired in CIBP. We also found the pain and motor behavioral differences in models that severed the patellar ligament vs. maintaining the patellar ligament. These findings provide a novel clue for further investigating the mechanisms responsible for the generation and development of CIBP.

## Introduction

Bone metastasis occurs in the later stages of most tumors, and in 70% of advanced breast and prostate cancers (Coleman, 2006). Bone metastasis often causes severe pain in patients with tumors. It is reported that 60–90% of patients with advanced cancer suffer from varying degrees of pain, of which about 30% suffer from persistent severe pain (Turabi and Plunkett, 2012).

The main symptom of bone metastasis is pain (von Moos et al., 2016; Macedo et al., 2017), moreover the pain get worse when patients use a limb affected by cancer (Jimenez-Andrade et al., 2010). Most papers have focused on the pain in the cancer induced bone pain (CIBP) model (Yang et al., 2018; Wang et al., 2021), however, the motor ability impairment by the enhancement of pain in the CIBP model is understudied. Here, we present a CIBP model by injecting Lewis lung cancer cells into the femoral medulla cavity. The mechanical, thermal, and cold allodynia as well as motor behaviors were tested. Interestingly, we found that the motor coordination and balance were severely impaired as CIBP increased.

Moreover, researchers usually sever the patellar ligament before injecting Lewis lung cancer cells into the femoral medulla cavity during the operation (Majuta et al., 2017; Wang et al., 2021). We suspect that severing the patellar ligament may affect paw withdrawal when we test pain behavior and movement. Therefore, we also reported the behavioral difference from pain and movement between severing or maintaining the patellar ligament.

## Materials and Methods

### Animals

This study was approved by the Animal Care and Use Committee of Nanjing University of Chinese Medicine (Nanjing, China). Experiments were conducted according to the animal research ethical guidelines of the International Association for the Study of Pain. Male C57BL/6 mice of 8 weeks old were used in our experiments. They were housed in groups of four per cage in the animal center of Nanjing University of Chinese Medicine, with free access to food and water and with a controlled temperature (22 ± 4°C), under an automatically controlled light cycle (light on 06:00–18:00 h). Only healthy animals weighing 20–25 g and displaying normal water and food intake were included in the study.

### Preparation of Lewis Lung Cancer Cell Line

Lewis lung cancer cell line (LLC) was obtained from the Cell bank of the Chinese Academy of Sciences (TCM7, Shanghai, China). Lewis cancer cells were washed with phosphate buffered saline (PBS). The cells were trypsinized using 0.05% trypsin at 37°C in the incubator for 2 min until the cells were fully dissolved. Media (DMEM high glucose with 10% fetal bovine serum and 5% Penicillin-Streptomycin) was added to end digestion. Suspended cells were then centrifuged 5 min at 1,000 rpm, and the media removed and cells re-suspended in 10 μl of fresh media. The cells were counted by a cell counter, and 1 × 10^6^ cells in 10 μl media of medium were injected into the medullary cavity of femur. Heat inactivated Lewis lung cancer cells were obtained by incubating at 50°C for 40 mins as done previously (Mao-Ying et al., 2006; Huang et al., 2008).

### Cancer Induced Bone Pain Model

Mice were anesthetized by intraperitoneal injection 1% pentobarbital sodium (50 mg/kg). After anesthesia, the eyes of the mice were smeared with vet ointment using a cotton swab to prevent dry eyes. The mouse was placed in a supine position on a fixed plate and the surgical limb (right limb) in a free position. The site of surgery was cleaned with 75% ethanol and the knee area was shaved. Using a scalpel, a longitudinal incision was made (about 0.5 cm) in the skin on the surface of the knee joint, exposing the knee joint. We then disconnected the patellar ligament (or it was left uncut for comparing behavior) and exposed the intercondylar fossa. An electric drill (P-500-10A, Guang Zhou, China) was used to make a hole in the intercondylar fossa along the direction of the femur. Then 10 μl of lung cancer cells (1 × 10^6^ cells in 10 μl media) were slowly injected into the medullary cavity by micro-syringe. There were three groups for pain test: control group (normal mouse without surgery), HIL group (injection of heat inactivated Lewis lung cancer cells into the medullary cavity) and CIBP group (injection of lung cancer cells into the medullary cavity). After surgery, the needle hole was sealed with bone wax and monitored for 2 min to prevent the lung cancer cells from leaking (Sasaki et al., 2015). After stitching the wound, the surgical area was sterilized and roxithromycin ointment was applied to the surgical area. The mouse was then placed in a clean cage in a supine position. Animals post-surgery were attended and monitored until they recovered from anesthesia.

### Behavioral Test

The behavioral assays were performed with the personnel blinded to different groups. All the mice were habituated to the room environment for 2 days before CIBP to measure the pain behaviors (baseline).

### Von-Frey Test

Animals were acclimated on an elevated metal grid (100 cm × 50 cm) for 30 min before the start of behavioral tests. Mechanical allodynia was recorded by measuring the paw withdrawal threshold with a set of Aesthesio Von Frey filaments (0.04–2 g; Ugo Basile, Gemonio, Italy). Von-Frey filament was applied to the plantar surface at a vertical angle for up to 3 s from the bottom when it bent to 90°. When the poke of one filament cannot induce a paw withdraw, an adjacent larger scale stimulus should be given. Fifty percent mechanical withdraw threshold values were determined by using the up-down method as described before (Chaplan et al., 1994).

### Cold Plate Tests

Mice were acclimated to the testing environment for 30 min before the initiation of cold plate tests. Then the mouse was placed on a cold plate device after the plate cooled down to 4°C. This device consists of three parts: the lid, the testing chamber, and the cube box. The testing chamber is 405 mm long, 122 mm in diameter. The cube box was 250 mm × 280 mm × 150 mm. Foot lifting was considered a positive reaction. The number of foot lifting reactions for mice within 5 min was the cold pain response index which is the cold pain threshold of the mouse as we have done previously (Ruan et al., 2018).

### Hargreaves Test

Thermal hyperalgesia was assessed by measuring the paw withdrawal latency to radiant heat stimuli. Mice were placed under a transparent plastic box (4.5 cm × 3 cm × 10 cm) on a glass platform (Plantar Test, 37370; Ugo Basile, Gemonio, Italy) and were allowed to acclimate for 30 min. The radiant heat source was applied to the center of the plantar surface of the hind paw with intervals of at least 3 min between applications. The radiant heat source and digital timer were activated. When the mouse withdrew its paw, the timer and heat source were stopped, determining the paw withdrawal latency time. Foot lift reaction was considered to be a positive reaction of the mouse to the stimulation. Each mouse was measured 3 times, average foot lifting latency time caused by thermal stimulation was recorded as the thermal withdrawal latency. The Hargreaves tests were measured as described previously (Hargreaves et al., 1988; Wang et al., 2019).

### Open Field Tests

This behavioral test used an open field device with a volume of 40 cm × 40 cm × 25 cm. The animal was placed in the central area of the open field box. The open field ground is composed of several grids. The corresponding activity parameters of the animal (the walking distance) within 5 min were recorded. A spontaneous activity video analysis system (Shanghai Jiliang Software Technology Co., Ltd., China) was used to analyze the data.

### Rotarod Tests

Before the experiment, the mice were placed on a 4 r/min rotating rod apparatus (47600, Ugo Basile Srl, Italy) to adapt to 60 s. The mice were placed on the drum of the rotarod. We then recorded the time the mice stayed on the rod (the internal rod is accelerated to 40 r/min at the initial speed of 4 r/min in 300 s) until falling down. The average time in the two groups (patellar ligament cut and uncut) is the latency on the rotarod.

### CT Scanning

An X-ray irradiation scan of the leg of mice with a micro-CT scanner (SkyScan1176, Bruker, Germany) was used to record the bone destruction in mice after CIBP surgery.

### HE Staining

After being washed in PBS, tissues were equilibrated sequentially in 15 and 30% sucrose, embedded in optimum cutting temperature compound, and sectioned with a cryostat. A HE staining kit (G1120, Beijing Solarbio Science and Technology Co., Ltd) was used. Soak the tissues sequentially in absolute ethanol for 5 min, 95% ethanol for 2 min, 80% ethanol for 2 min, 70% ethanol for 2 min, distilled water 2 min. Dyeing with hematoxylin solution for 5–20 min, differentiation fluid 30 s, and immersed with tap water for 15 min. Eosin staining for 30 s−2 min, immersed with tap water for 5min. They were sealed with neutral gum and observed under microscopy (Axio Zoom.V16, Zeiss, Germany).

### Statistical Analysis

Two-way repeated measures ANOVA followed by *post-hoc* analysis (SPSS version 16.0) were used to compare the behavioral results. A value of *P* < 0.05 was considered statistically significant in all tests.

## Results

### CIBP Surgery Induced Significant Mechanical, Heat, and Cold Allodynia

**Figure 1:**
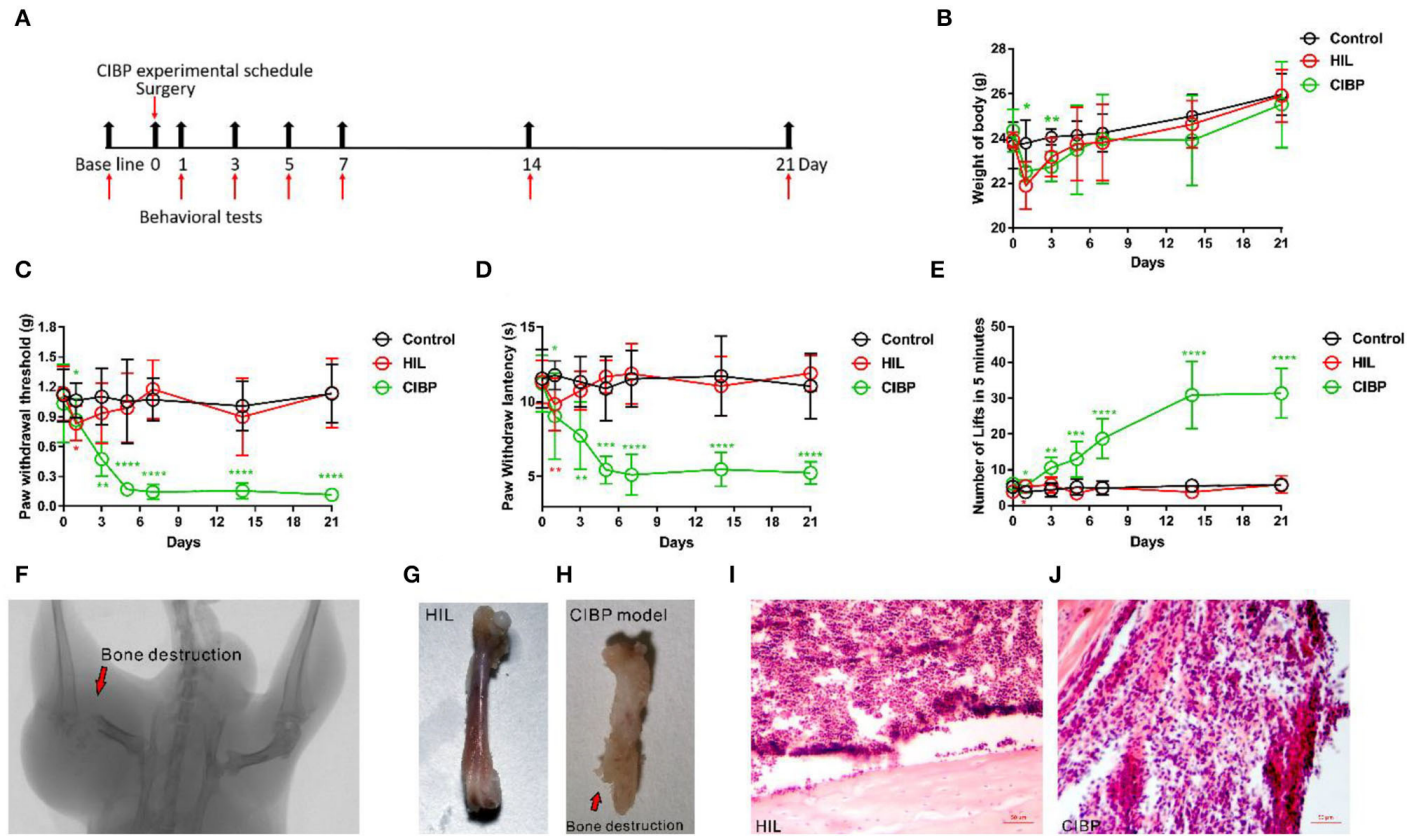
The mechanical, heat, and cold allodynia enhanced after injecting Lewis lung cancer cells. **(A)** Experimental schedule of the CIBP model. **(B)** The weights were recorded after CIBP operation, *n* = 6 mice/group. **(C)** The mechanical allodynia was tested after CIBP surgery, *n* = 9 mice/group. **(D)** The heat allodynia was recorded after CIBP operation, *n* = 9 mice/group. **(E)** The cold allodynia was recorded after CIBP operation, *n* = 9 mice/group. **(F)** Radiograph results represent robust radiolucent lesions (arrowhead) of the femur on the 21st day. **(G,H)** The femur is intact in the control group while the bone shows fractures and holes swallowed by Lewis lung cancer cells in the CIBP group on the 21st day. **(I,J)** Histopathological sections (hematoxylin and eosin stain) show that the bone marrow was largely replaced by invading tumor cells with medullary bone loss and femur destruction on the 21st day. Scale bar: 50 μm. ^*^p < 0.05, ^**^p < 0.01, ^***^ *p* < 0.001, ^****^ *P* < *0.0*001 (*in red font is compared between HIL and control group, *in green font is compared between CIBP and HIL group), two-way **(B–E)** repeated measures ANOVA followed by *post-hoc* analysis.

We first established a mouse model of CIBP by injecting Lewis lung cancer cells into the bone marrow cavity of the femur. Weight and pain behavior were measured after CIBP surgery as outlined in the experimental procedure (Figure 1A). The weight of the control group (without surgery), HIL group (injected with heat inactivated Lewis lung cancer cells), and CIBP group (injected with Lewis lung cancer cells) were compared. We found the weight of the HIL group was lower than the control group in the first and third day after the operation, but there was no difference after the fifth day (Figure 1B). This most likely pointed to the initial surgery as the leading factor in weight loss. We did not detect any weight differences among the three groups after the fifth day, although the CIBP group did trend lower. We then measured mechanical, heat, and cold allodynia after the CIBP operation. The mechanical, heat and cold allodynia were increased significantly compared with the HIL group after CIBP operation (Figures 1C–E).

Using a CT scan, we found significant destruction in the femur from mice that had undergone CIBP surgery by X-ray irradiation, while the negative control side was largely intact after HIL injection (Figure 1F). The fractures usually appeared 14 days after CIBP surgery. We harvested the bone 21 days after surgery and found noticeable bone destruction in the CIBP model (Figures 1G,H). The hematoxylin and eosin-stained femur sections showed both tumor growth and bone destruction with medullary bone loss on the 21st day (Figures 1I,J). In conclusion, we showed that injection of Lewis lung cancer cells can induce significant pain behaviors and provide a consistent and repeatable model for CIBP.

### Motor Coordination and Balance Were Impaired in Mice With Cancer Induced Bone Pain

**Figure 2:**
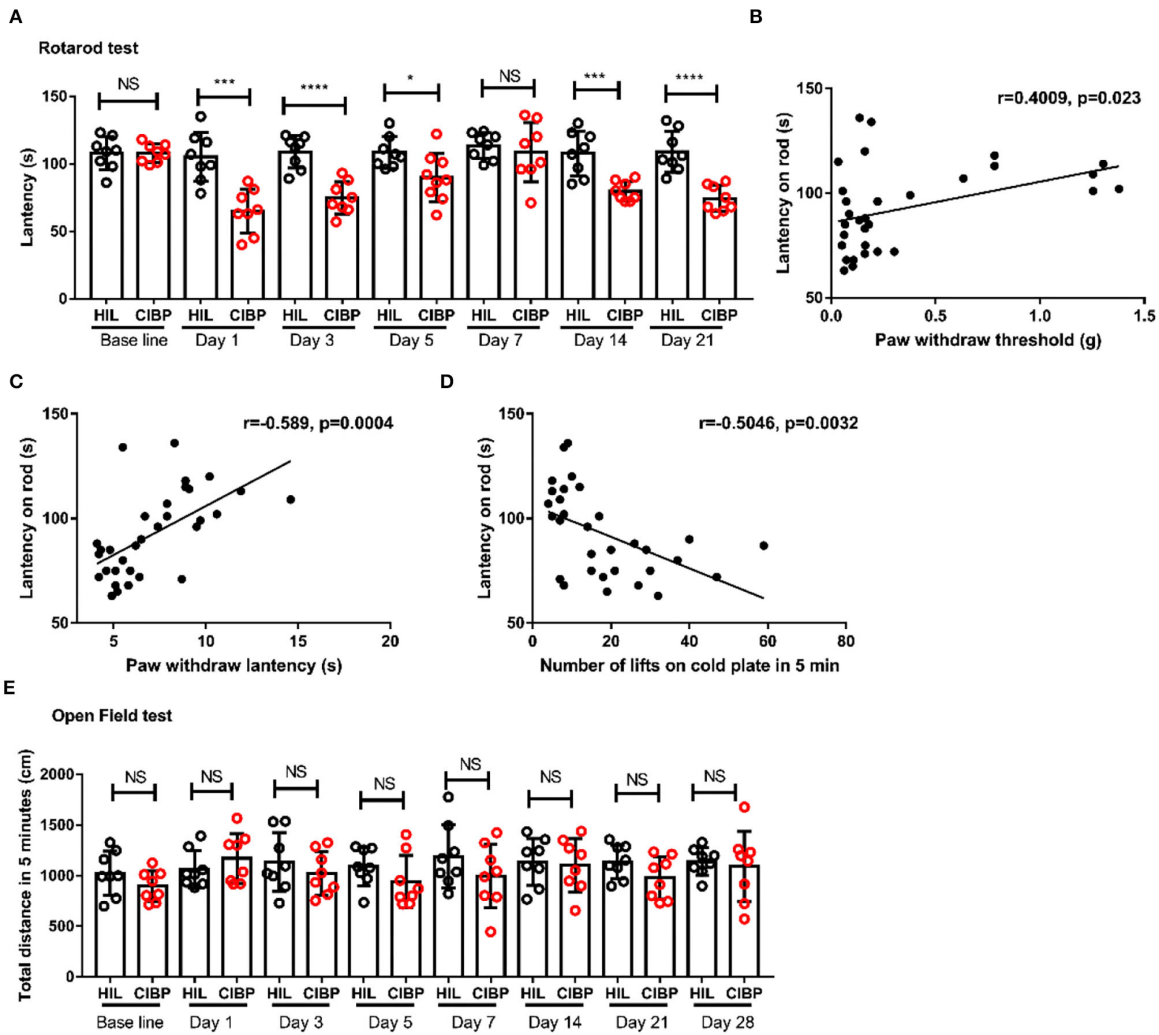
Motor coordination and balance were impaired in mice with CIBP surgery. **(A)** The latency in rotarod tests between the HIL and CIBP groups. *n* = 8 mice/group. **(B)** Linear analysis of motor coordination with mechanical allodynia. **(C)** Linear analysis of motor coordination with hot allodynia. **(D)** Linear analysis of motor coordination with cold allodynia. **(E)** The motion distance compared between the HIL and CIBP group in the open field test. *n* = 8 mice/group. *P < 0.05, *** *P* < 0.001, ****P < 0.0001 by two-way **(A,E)** repeated measures ANOVA followed by *post-hoc* analysis.

To investigate the locomotor activity and motor coordination followed by CIBP, open field and rotarod tests were applied to HIL and CIBP group mice. Interestingly, we found the latency staying on the rod significantly reduced in CIBP group on the 1st, 3rd, 5th, 14th, and 21st day (Figure 2A). The decline on the 1st, 3rd, and 5th day may be due to CIBP surgery. The decline of latency after the 14th day is most likely due to CIBP as the tumor grew and pain increases. This result showed that the motor coordination and balance were impaired due to CIBP after a correlation analysis (Figures 2B–D). However, we didn't find any difference between the HIL group and the CIBP group in the open field test (the amount of time spent close to the wall, time spent, and number of entries into the center zone are not shown). The total distance in 5 min were similar between the two groups (Figure 2E). Together, these results suggested that the motor coordination and balance were impaired following cancer induced bone pain.

### Cutting off the Patellar Ligament Did Not Affect Movement or Pain Behavior in a Mice Model of CIBP

**Figure 3:**
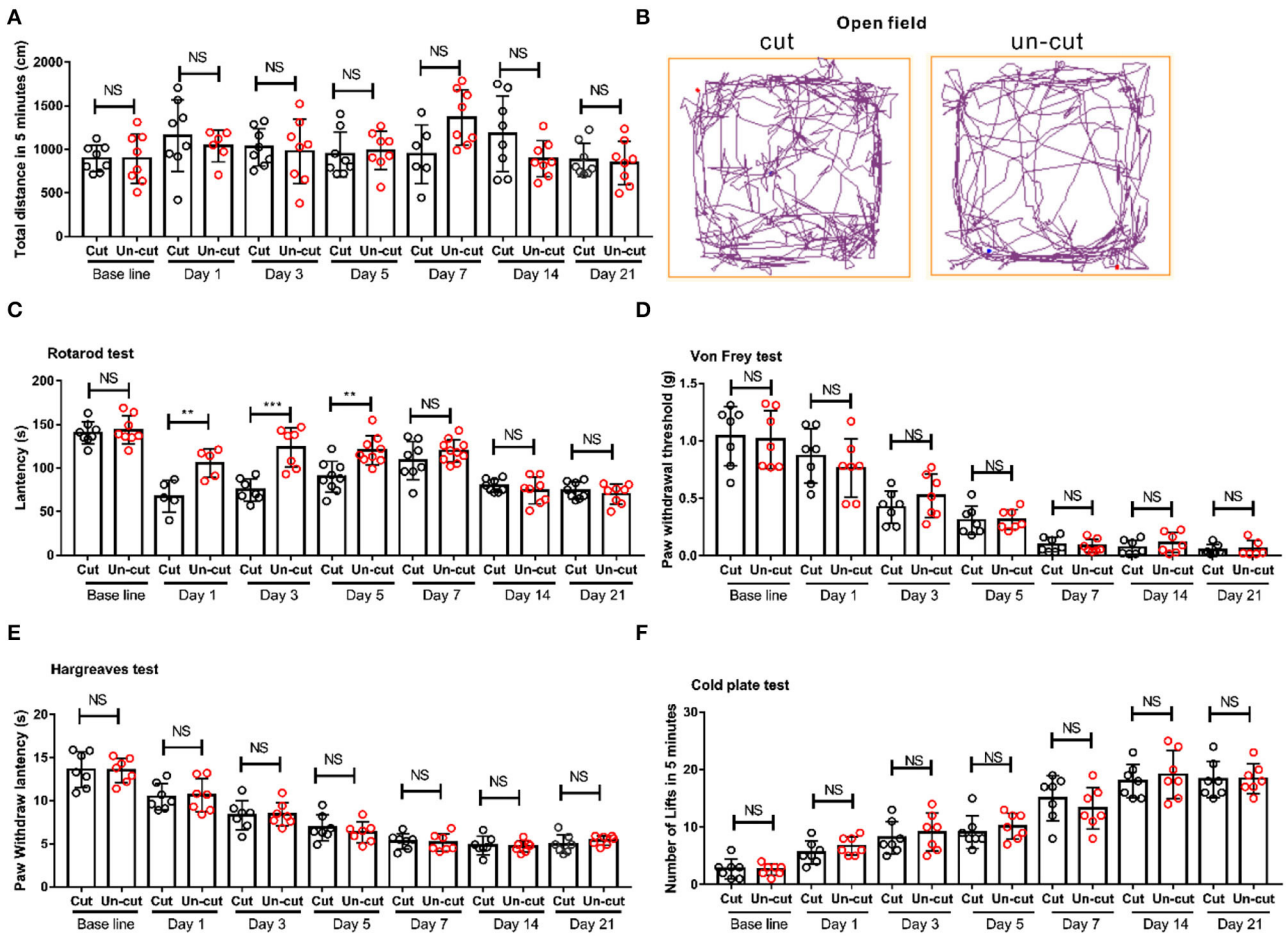
The behavioral difference in outcomes between severing or maintaining the patellar ligament. **(A)** The motion distance compared between patellar ligament cutting off and patellar ligament uncutting off group on the base line, 1st, 3rd, 5th, 7th, 14th, and 21st day. *n* = 8 mice/group. **(B)** Representative pictures show movement track of mice within 5 mins in patellar ligament cutting off and patellar ligament un-cutting off group in the open field tests on the 21st day after CIBP surgery. **(C)** The latency in rotarod tests of the two groups on base line, 1st, 3rd, 5th, 7th, 14th, and 21st day. *n* = 8 mice/group. **(D)** Von-Frey test results of the two groups, *n* = 7 mice/group. **(E)** Thermal radiation test results of the two groups, *n* = 7 mice/group. **(F)** Cold plate test results of the two groups, *n* = 7 mice/group. **P < 0.01, *** *P* < 0.001 by two-way **(A,E)** repeated measures ANOVA followed by *post-hoc* analysis.

Cancer induced bone pain models usually cut the patellar ligament (Schwei et al., 1999; Sasaki et al., 2015). To assess whether severing the patellar ligament affects paw lifting behavior, we measured and compared the motor coordination and pain behaviors in CIBP models with and without severing the patellar ligament. We recorded and compared the movements of mice subjected to ligament cutting or not by an open field test (Figures 3A,B). The movements before operation (baseline), 1st, 3rd, 5th, 7th, 14th, and 21st day after the CIBP operation were recorded. We found no difference between the two groups (Figure 3A). After the 14th day, the mouse usually walked on its front toes, but the walking distance was the same on the 14th and 21st day (Figure 3B). To detect the coordination and balance, the latency on the rotarod between mice that had undergone cutting off the ligament group vs the uncutting off group were compared. The latency on the rotarod was reduced in the group with the severed ligament on the 1st, 3rd, and 5th day, while the latency was the same at base line, on the 7th, 14th, and 21st day (Figure 3C). The reduction from the 1st to the 5th day may be due to the cutting of the patellar ligament. Despite the observation that mice walked on their front toes on the 21st day, the latency on the rotarod was not affected after the 14th day (Figure 3C). The mechanical, heat, and cold allodynia were also compared between the two groups. We found cutting of the patellar ligament did not affect the pain behavior compared to the HIL group in the CIBP model (Figures 3D–F). The above results confirmed that cutting off the patellar ligament did not affect movement or pain behavior in a mice model of CIBP.

## Discussion

To investigate the mechanisms of CIBP, a mouse model of CIBP was proposed by Schwei in the 1990s (Schwei et al., 1999). This CIBP model is well-established (Pineda-Farias et al., 2020), but the motor ability after the initiation of the CIBP model is under studied. To improve the quality of life from those that suffer from CIBP, the study of CIBP induced movement impairment is important. Here, we established a mouse model of Lewis lung cancer cells that induced CIBP and found an impairment of motor coordination and balance concomitant with enhanced pain.

The main symptom in the CIBP model is allodynia. Thus, we tested mechanical, heat, and cold pain to confirm whether this model leads to increases in allodynia. Results suggest that the CIBP model is associated with an increase in peripheral afferent hypersensitivity. Behaviorally, mice in the HIL group and control group showed no signs of pain during palpation of the hind paw. However, injection with live lung cancer cells resulted in significant increases in pain. Furthermore, severe pain behavior was observed in mice as the bone destruction occurred. Nevertheless, we believe that cancer induced bone pain is correlated with bone fracture after the 14th day (Luger et al., 2005; Wakabayashi et al., 2018). This phenomenon is the same as bone metastasis of lung cancer found in clinical reports (Serkan et al., 2019). Bone cancer destruction leads to touch, heat, or cold evoked pain behaviors, therefore, most researchers use this cancer induced bone pain model to study the mechanism of cancer induced bone pain (Hansen et al., 2011; Fang et al., 2015; Fuseya et al., 2016).

Although movement evoked pain is an important factor as in CIBP, motor impairment in CIBP has been ignored in past research. To reveal the motor ability changes due to severe pain, we examined the locomotor activity and motor coordination by using the open field test and rotarod test in CIBP mice. Interestingly, the latency on rod declined in CIBP mice while the distance in open filed test was unchanged. The results indicate that the motor coordination and balance were impaired seriously by CIBP as we did a correlation analysis between pain behavior and motor behavior. The finding that locomotor activity remained unchanged in the open field test recapitulates findings from past groups (Majuta et al., 2017). There are probably two reasons for this phenomenon. First, the CIBP impairs the motor coordination, and therefore the latency on the rotarod test is reduced, but the relatively mild exercise in the open field test was not affected. Secondly, there is a “bridge” between pain and locomotor activity (Campbell et al., 2015). Neuropeptides play a central role in the regulation of sense and motor pathways, dysregulation of neuropeptide processing affects the sensory system and motor behavior (Campbell et al., 2015). We suspect peptidergic abnormalities impaired the motor coordination found in CIBP mice and further research is certainly warranted.

Severing the patellar ligament makes the operation much easier. More importantly, we compared the exercise behavior and pain behavior between cutting or maintaining the patellar ligament as we suspected that severing the patellar ligament affects behavior. The motor coordination decreased in the severed group from the 1st to the 5th day. This may be due to the surgery affecting motor ability. However, the decrease disappeared after the 7th day when the mice recovered from surgery. We found that mice usually don't use the injured paw when running on the rotarod when the tumor is most obvious after the 14th day. The pain behaviors were found the same between the two groups. This may be due to the repair ability in mice. Therefore, we believe that severing the patellar ligament does not affect the pain behavior.

Our results revealed motor coordination impairment in CIBP and answered several questions about the CIBP model. This study provides a novel clue for further investigating the mechanisms responsible for the generation and development of CIBP.

## Data Availability Statement

The raw data supporting the conclusions of this article will be made available by the authors, without undue reservation.

## Ethics Statement

This study was approved by the Animal Care and Use Committee of Nanjing University of Chinese Medicine (Nanjing, China). Experiments were conducted according to the Animal Research Ethical Guidelines of the International Association for the Study of Pain.

## Author Contributions

CW conceived and designed the project. HJ and XJ carried out the experiments. CW and GY reviewed and performed the statistical analyses. QZ, YZ, CZ, and YY helped breed the animals, participated in animal models, and genotyping experiments. CW, ZT, and GY reviewed and finished the manuscript. All authors read and approved the final manuscript.

## Funding

This work was supported by the National Science Foundation of China to CW (81600966) and the Natural Science Foundation of Jiangsu Province to CW (BK20161042).

## Conflict of Interest

The authors declare that the research was conducted in the absence of any commercial or financial relationships that could be construed as a potential conflict of interest.

## Publisher's Note

All claims expressed in this article are solely those of the authors and do not necessarily represent those of their affiliated organizations, or those of the publisher, the editors and the reviewers. Any product that may be evaluated in this article, or claim that may be made by its manufacturer, is not guaranteed or endorsed by the publisher.
